# Safety maximization of percutaneous transthoracic needle biopsy with ultrasound guide in subpleural lesions in the evaluation of pulmonary consolidation

**DOI:** 10.1186/s12931-019-1031-0

**Published:** 2019-04-05

**Authors:** Marco Sperandeo, Anna Del Colle, Elisabettamaria Frongillo, Gaetano Rea, Lucia Dimitri, Cristiana Cipriani, Donato Lacedonia

**Affiliations:** 1 Unit of Interventional and Diagnostic Ultrasound of Internal Medicine IRCCS Fondazione Casa Sollievo della Sofferenza, San Giovanni Rotondo, Italy; 2 Department of Medical and Surgical Sciences, Institute of Respiratory Disease, University of Foggia, Viale degli Aviatori, 2, 71122 Foggia, FG Italy; 3 Unit of Thoracic Surgery IRCCS Fondazione Casa Sollievo della Sofferenza, San Giovanni Rotondo, Italy; 4 Department of Radiology, Ultrasound Diagnostic Unit, Monaldi Hospital, dei Colli, Naples, AO Italy; 5 Unit of Pathology IRCCS Fondazione Casa Sollievo della Sofferenza, San Giovanni Rotondo, Italy; 6 Department of Internal Medicine and Medical Discipline, Sapienza University of Rome, Rome, Italy

**Keywords:** Percutaneous transthoracic needle biopsy, Pulmonary consolidation, Subpleural, Transthoracic ultrasound

## Abstract

The study by Kiranantawat et al. “Clinical role, safety and diagnostic accuracy of percutaneous transthoracic needle biopsy in the evaluation of pulmonary consolidation” highlights how “pulmonary consolidation can be safely evaluated with CT-guided percutaneous needle biopsy”. Even if we agree about the role of CT guidance, we would like to point out how Thoracic Ultrasound could be better than CT for biopsy of subpleural lesions that could easily be detected and reached with this “real-time” and quicker technique.

Dear Editor,

We read with great interest the article by Kiranantawat et al. [1], recently published in your journal, focused on the correct management of gaining access to the pulmonary consolidations. Their statements are valid and easily transferable to clinical practice. As perfectly explained in their article, pulmonary consolidation is a very common clinical finding that often put the clinician in front of a hard differential diagnosis. Usually the first etiological diagnostic attempt is a bronchoscopy with bronchoalveolar lavage but sometimes this is not enough to have a definitive diagnosis. Therefore, the Authors showed how Computed Tomography (CT)-guided percutaneous needle biopsy (PTNB) could be a very good and useful option to have additional tissue sample in a safe and accurate way. However, according to us, the Authors should have better highlighted the roles of CT guidance for lung consolidations and the available alternative diagnostic tools. Indeed, the authors did not mention the opportunity to perform transthoracic ultrasound (TUS)- guided biopsies. We agree with CT-guided technique when consolidation is deep and could not be correctly identified by TUS, but it is mandatory to underline that ultrasound has to be considered a valuable tool in detecting even small lesions adherent to the pleural surface [2, 3]. The procedure-related complications with TUS guidance are less frequent than reported in the review [4]. In our experience, in a case series [5], including 95 ultrasound-guided PTNB, there was no pneumothorax or hemothorax. A further case series of 801 ultrasound-guided PTNB reported [6] four subtotal pneumothorax, all spontaneously resolved, and no hemorrhagic pleural effusion. In all these cases, “modified Menghini” technique was used. It is the same procedure employed in hepatic biopsies and it consists of a needle with stylet connected to a syringe plunger, where the needle tip is a Menghini type tip, whereas the stylet tip is pyramidal. The needle (18 gauge) is labeled with a centimeter scale to have the highest precision level and the device diameter is identified by an international color code. This provides specimens suitable for histologic diagnosis and minimizes the occurrence of complications, which appear to be more frequent with needles of higher caliber (14–16 gauge) needles [7]. It is advisable the use of a dedicated probe with a central hole through which the needle set is introduced [5] (Fig. 1). This is still the most suitable and reliable approach for these purposes because the needle is visible in real time during the entire procedure, thanks to Ultrasound machine. Compared to CT, in appropriate cases, TUS guidance is safe, inexpensive, rapid, easy to have in every department of all hospitals and easy to use; in addition, there is no ionizing radiation exposure issue [8]. Moreover, TUS guidance is easily avaiable at patient bedside and this could be extremely useful in patients with low performance status. Thus, the use of ultrasound guidance is an important aid to better perform a variety of diagnostic transthoracic interventions for chest wall, lung parenchymal and pleural pathology. In conclusion, we really think that ultrasound guidance is a better option both for the patient both for the clinician to guide invasive procedures with the aim of having pleural and lung consolidations samples. TUS-guided PTNB should be the “first choice” as a diagnostic procedure in pleural and adherent to pleural pulmonary lesions [9].

**Fig. 1:**
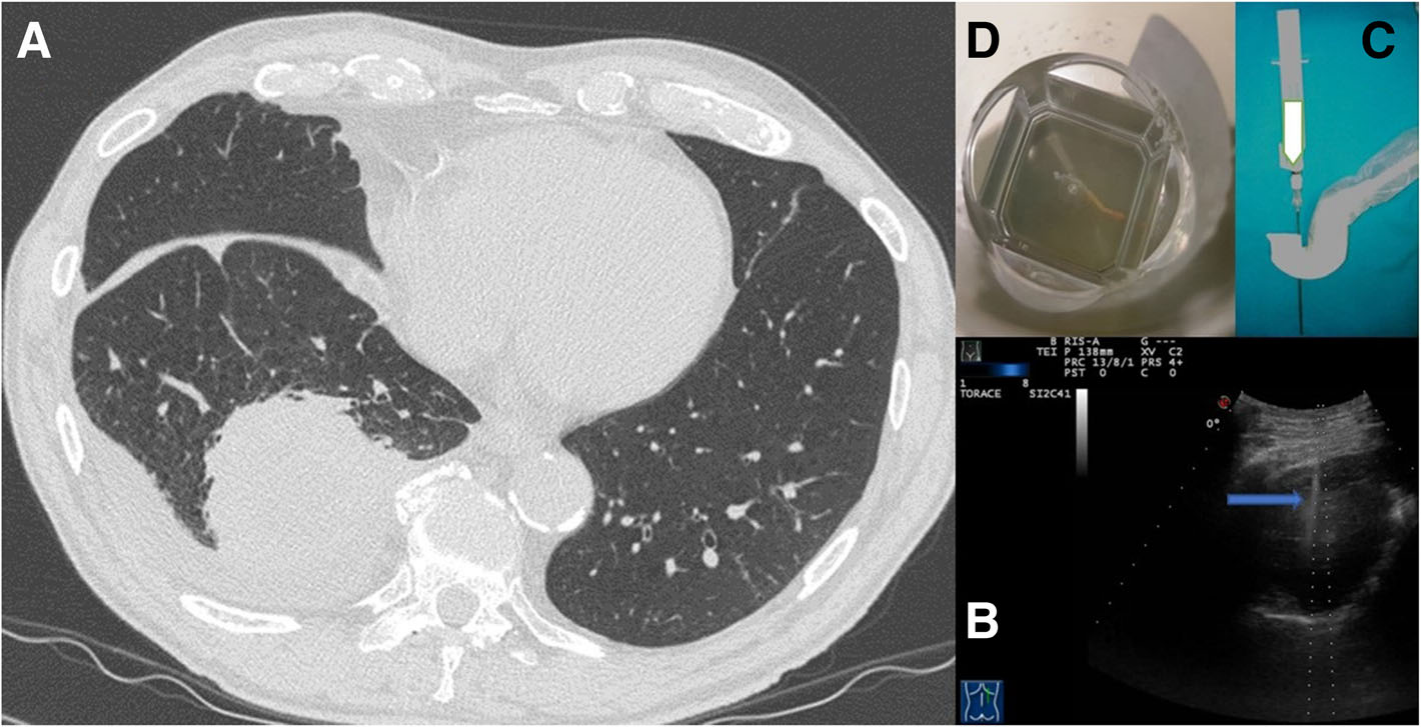
**a** Axial chest computed tomographic (CT) image detected a solid lung nodule suggestive of malignancy in the periphery of the right middle lobe. This lesion has broad pleural contact. **b** The same lung nodule seen during Ultrasound guided biopsy. We can see the needle (blue arrow) within the consolidation in the right middle lobe.**c** A dedicated probe with a central hole through which the needle set is introduced. **d** Specimen suitable for histologic diagnosis (adenocarcinoma)

## Abbreviations

CT: Computerized Tomography
PTNB: Percutaneous transthoracic needle biopsy
TUS: Thoracic Ultrasound
